# *Staphylococcus aureus* forms spreading dendrites that have characteristics of active motility

**DOI:** 10.1038/srep17698

**Published:** 2015-12-18

**Authors:** Eric J. G. Pollitt, Shanika A. Crusz, Stephen P. Diggle

**Affiliations:** 1 School of Life Sciences, University Park, University of Nottingham, Nottingham, NG7 2RD, U.K; 2 Department of Molecular Biology and Biotechnology, Firth Court, University of Sheffield, Sheffield, S10 2TN, UK

## Abstract

*Staphylococcus aureus* is historically regarded as a non-motile organism. More recently it has been shown that *S. aureus* can passively move across agar surfaces in a process called spreading. We re-analysed spreading motility using a modified assay and focused on observing the formation of dendrites: branching structures that emerge from the central colony. We discovered that *S. aureus* can spread across the surface of media in structures that we term ‘comets’, which advance outwards and precede the formation of dendrites. We observed comets in a diverse selection of *S. aureus* isolates and they exhibit the following behaviours: (1) They consist of phenotypically distinct cores of cells that move forward and seed other *S. aureus* cells behind them forming a comet ‘tail’; (2) they move when other cells in the comet tail have stopped moving; (3) the comet core is held together by a matrix of slime; and (4) the comets etch trails in the agar as they move forwards. Comets are not consistent with spreading motility or other forms of passive motility. Comet behaviour does share many similarities with a form of active motility known as gliding. Our observations therefore suggest that *S. aureus* is actively motile under certain conditions.

Motility is central to a number of bacterial behaviours, such as biofilm formation, virulence, and host colonization^1^,^2^,^3^. Consequently, motility is one of the key characteristics that can be used to determine the range of possible behaviours of a given bacterial species. *Staphylococcus aureus* is historically defined as a non-motile organism^4^, but here we present observations that show it engages in a behaviour that is consistent with it being actively motile under certain conditions.

It has been previously demonstrated that colonies of *S. aureus* can passively expand across the surface of soft agar plates, aided by the production of Phenol Soluble Modulin (PSM) surfactants, in a process called spreading^5^,^6^. This can be more accurately defined as a form of passive motility called sliding. Sliding is defined by the propulsive force being provided by growth of the bacterial cells within the colony forcing each other outwards, and which is aided by surfactant production, which prevents the bacteria getting stuck to the surface on which they are moving^3^,^7^,^8^. Organisms that only engage in passive motilities such as sliding, are defined as non-motile, due to a lack of obvious motility mechanisms such as flagella^9^. Passive movement has been defined to include: sliding, darting, carriage by fluid, or Brownian motion^3^,^10^. Darting is where bacterial cells overcome, through growth, the adhesive forces holding them to other cells and sporadically eject themselves short distances. If enough fluid flow is present, this can also move bacterial cells across a surface^10^. Passively motile bacteria move either in a random limited fashion (darting, brownian motion), or all the mobile bacteria present move in the same way simultaneously over a broad front, due to the application of relatively powerful external forces that overcome surface adherence (sliding, carriage by fluid).

Actively motile bacteria are distinguished from passively motile bacteria via two logical arguments: either by, (1) observing previously identified characteristics that are associated with active motility and not passive motility (e.g. flagella), or (2) identifying situations where active propulsion can only be responsible for the observed movement of the bacteria (e.g. observing that the bacteria swim). Active motility includes swimming, swarming, twitching and gliding^3^. Swimming is dependent on flagella, whilst swarming occurs when groups of hyper-flagellated bacteria move together across surfaces. Twitching is dependent on the extension and retraction of type IV pili. Gliding motility is observationally defined as the continuous smooth movement of the bacteria, either singly or as groups of cells in either a linear or a whirling pattern^3^,^11^,^12^. It has evolved in a diverse range of bacterial genera such as *Myxococcus*, *Cyanobacteria*, *Cytophaga*, *Mycoplasma* and *Beggiatoa*^9^,^11^,^12^,^13^. Gliding is theorized to be powered by a range of possible mechanisms (depending on the bacterial species involved), which include slime extrusion, focal adhesion complexes, cell twisting, type IV pili or membrane protrusion^9^,^11^,^13^,^14^. Although the mechanism of movement differs and remains to be fully elucidated in many bacterial species (due to lack of observable surface structures), several further characteristics have been identified that are associated with gliding. These are: (1) a requirement for a solid surface on which to move; (2) an absence of flagella; (3) production of slime (defined here as disorganized matrix of extracellular material) around or next to the gliding bacteria, and (4) the formation of tracks where the gliding bacteria have either etched into a solid surface or left behind a trail of slime^3^,^11^,^12^. More broadly, actively moving bacteria tend to share the following observable characteristics: they engage in directed discrete movement either as individual or groups of cells (not all bacteria are moving in the same way at the same time) and slime production is broadly associated with bacteria moving on surfaces (swarming and gliding motility)^3^,^15^,^16^.

Previous studies of spreading motility in *S. aureus* have not experimentally investigated the finger-like dendrites that can be observed emerging from the central colony^5^,^6^,^17^,^18^,^19^, and so here we examine the movement of *S. aureus* across the surface of specially modified motility plates, with a focus on investigating dendrite formation in *S. aureus*. We first show that dendrite formation is a behaviour maintained in diverse *S. aureus* strains (as defined by their differing quorum sensing *agr* types)^20^. We then show that these dendrites are preceded by ‘comets’, structures that move outwards from the center of the colony, seeding cells behind them, which then grow into observable dendrites. After 8 h of colony expansion, the comet heads are the main source of movement. Cells in the tail follow these comet heads for a period whilst bacteria further away no longer move. Comet movement is apparently reliant, to some extent, on surfactant production. Comet heads are composed of aggregates of *S. aureus* cells held together by a matrix of slime and display no observable pili or flagella. Under certain conditions, comets can also etch the agar, leaving behind tracks. We then show that the moving *S. aureus* colonies are capable of avoiding other colonies, and that the colonies are surrounded by surfactant. We finally show that the addition of exogenous fluid is not able to effectively move comet heads, but can easily move the cells in the comet ‘tails’. Overall we demonstrate that these behaviours are consistent with active motility, and most closely resemble gliding motility.

## Results

### *S. aureus* colony spreading on revised motility assay plates

**Figure 1:**
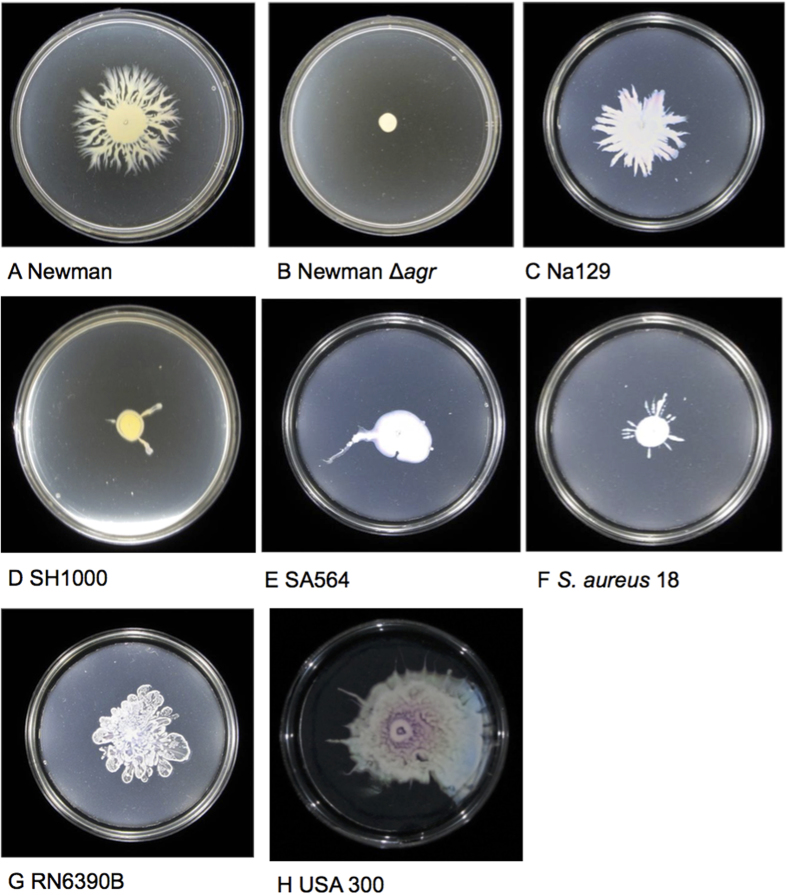
Expansion of *S. aureus* in a modified motility assay. This assay produced similar results to a previously described spreading assay and showed *S. aureus* can spread radially across low agar plates in 9 cm diameter Petri dishes when incubated at 37 °C. Movement is conserved in strains from diverse *agr* types (the *agr* type of each strain is detailed in Table 1). All the wild type strains tested produced dendrites apart from RN6390B, and Newman consistently produced the most dendrites. *agr* mutants do not move probably because they do not produce surfactant. The presented images are representative of 3 independent biological replicates.

In common with the previously described spreading assay^5^, spotting *S. aureus* culture onto the center of plates made using a modified assay (see Materials and Methods), results in wild-type *S. aureus* spreading radially across motility agar. Using this assay, we found that finger-like dendrites are formed, and that the motility behaviour is *agr-*dependent (Fig. 1)^6^,^18^. The Newman isolate made the most dendrites, but dendrites occurred in most strains tested (apart from RN6390B). The colonies typically stopped expanding after 24 h when the plates had been incubated at 37 °C.

### Dendrites are preceded by ‘comets’ of *S. aureus* cells

**Figure 2:**
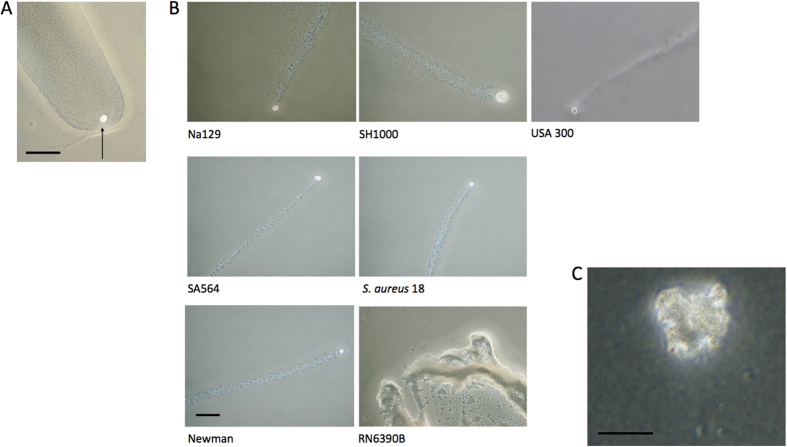
“Comets” of *S. aureus* cells form in front of dendrites. Dendrites were observed using phase contrast microscopy (**A)**. After 15 h growth, phase bright aggregates (indicated with an arrow) occurred at the tip of the dendrites (Newman strain shown) Scale bar = 100 μm; (**B**) After 8–12 h of incubation in all the strains that produced dendrites, dendrites were preceded by ‘comets’ of cells, a phase bright aggregate that had a trail of cells behind it leading back to the central colony (at ×100 magnification). Only RN6390B did not produce comets. All images are at the same scale, Scale bar = 100 μm; (**C**) The Newman strain comet head at ×400 magnification on a 10 ml plate. The image shows that the phase bright comet cores are composed of a grouping of cells, Scale bar = 20 μm. All presented images are representative of 3 independent biological replicates.

We next examined the motility plates under a phase contrast microscope after 15 h of growth at 37 °C. We found that the tips of many dendrites contained a phase bright core of unknown composition, which usually disappeared after 24 h growth (Fig. 2A). We next examined the tips of dendrites on plates that had been incubated for 8–12 h and found that all the dendrites were preceded by structures that we colloquially term ‘comets’. These comets consisted of phase bright cores that were followed by trails of cells expanding from the main mass of the colony (Fig. 2B). Although the core shape could be irregular, they tended to be oval in shape. We observed that comet formation occurred across a range of *S. aureus* isolates of differing *agr* types (I–IV), and in particular the MRSA strain USA 300, although comet formation was not completely universal. For example, RN6390B did not make dendrites and consequently did not make comets, instead it produced a series of fan shaped structures, which we did not investigate further (Figs 1 and 2B). However, SH1000, a close relative of RN6390B^21^, did produce comets. We then examined comet formation on modified 10 ml motility plates at ×400 magnification and observed that the center of a comet core was composed of a heaped grouping of *S. aureus* cells, whereas the cells in the tail consisted of a monolayer of cells (Fig. 2C). The Newman strain produced the most dendrites and therefore the most comets (Fig. 1). The Newman strain was therefore used for all subsequent experiments.

### Time-lapse analysis of comet movement

**Figure 3:**
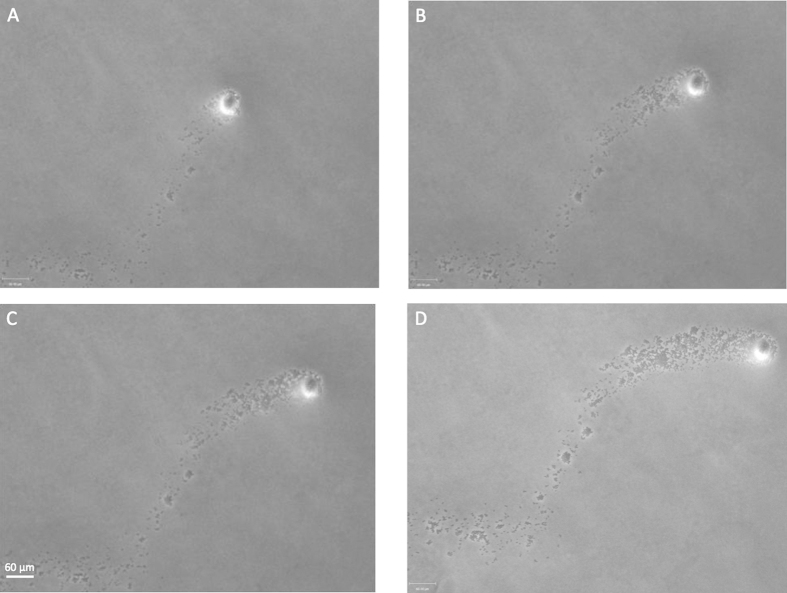
Movement of *S. aureus* in comets. These four photos show the same comet at different times, in sequence from (**A–D**) (summary photos of comet seen in Video S3). The main *S. aureus* colony is just below the bottom left of each image frame. The comet core is moving forwards and the bacteria in the comet tail largely do not move apart from growth *in situ* as a comparison of the images shows. Surfactant movement alone would not be able to move the comet head without disrupting the comet tail. Scale bar = 60 μm.

To classify motile behaviours, the bacterial movement in question needs to be observed over an extended timeframe^3^. We therefore examined dendrites using a microscope with a heated cabinet, and generated time-lapse videos. Initially after 5 h growth, no comets were present, but a behaviour consistent with sliding motility was observed, where all the cells in the field of vision are moving in the same direction on a wave of fluid (Video S1). Between 5 and 8 h post inoculation, a mix of comet formation and sliding occurred. In Video S2, comet formation and motility can initially be observed, but later, the comets are dispersed by sliding bacterial cells on a wave of surfactant (Video S2). After 8 h, we found cases where the only form of directional movement present was associated with the comet. We found that the comet clusters advance forward seeding other cells behind them without apparently losing any mass in the comet head (Fig. 3; Video S3). We also observed that the bacterial cells in comet tails were able to follow the comet tips for a period of time but this ability decreased the further away they were from the comet heads. The cells in the comet tip moved at speeds of between 230–1200 μm/h, with faster speeds associated with earlier observation times.

### *S. aureus* comet cores are held together by a slime matrix

**Figure 4:**
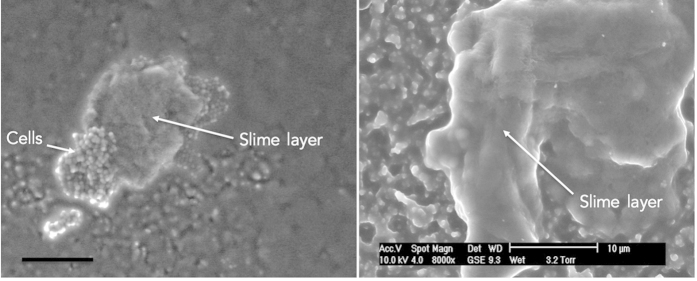
The comet cores are encased in a slime matrix. We used ESEM to observe the comets at high magnification without disrupting them. The comet cores are surrounded by a matrix of slime, which is consistent with the phase bright cores seen in Fig. 2. The left image shows the slime is associated with *S. aureus* cells (scale bar = 20 μm), whilst the right image shows a comet core completely surrounded by slime.

We observed the structure of the *S. aureus* comets using Environmental Scanning Electron Microscopy (ESEM) to complement the work performed with the light microscope. ESEM enabled us to observe the comets at high magnification without disrupting the comet structure^22^. We found that the cells that made up the comet core were surrounded by a matrix of slime (disorganized extracellular material deposited around the bacteria, the chemical composition is unknown) (Fig. 4; Fig. S1A). We observed no trail of slime behind the comet core (Fig. S1B) (slime trails occur in some gliding bacteria such as *M. xanthus* and *Beggiatoa*^15^). The production of slime was not observed anywhere else in the colony, with the *S. aureus* cells elsewhere closely resembling other observations of *S. aureus* made with ESEM (Fig. S1C). No pili or flagella-like structures were observed in the comet or anywhere else in the colony. We subsequently searched for homologues in *S. aureus* (using BLAST-P) of motility related proteins such as flagellin (core subunit of the flagella), pilin (the core subunit of type IV pili) and known global regulators associated with motility in other organisms. As expected, no homologues were found in *S. aureus* (data not shown)^23^.

### *S. aureus* comets leave physical tracks in agar

**Figure 5:**
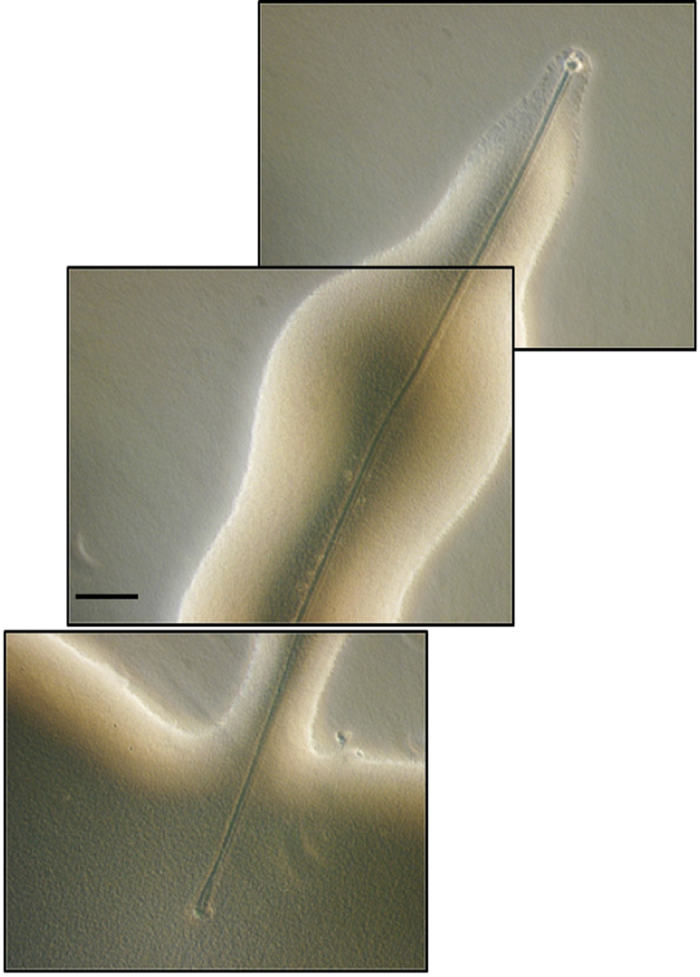
Comets can etch tracks in the agar. On 10 ml plates, the comets made tracks that we could observe with a phase contrast microscope. These tracks started within the main mass of the colony and always ended in a comet core. Scale bar = 100 μm.

Whilst modifying the assay for ESEM, we observed that tracks could be seen behind the comets on motility plates, where the colony had expanded to a portion of the plate where the nitrocellulose backing to the agar was not present, and the comets could be observed with a phase contrast microscope. We used the modified 10 ml plate assay to view the tracks because they were more easily observed on thinner agar (Fig. 5). The tracks originated within the central mass of the main *S. aureus* colony and always ended in a comet core. These tracks appear phase bright when no cells are present and phase dark when cells are present (Fig. S2A), and this appears to be consistent with the comets etching the agar as they move. Hypothetically the *S. aureus* comets can thin the agar as they move, resulting in the phase bright trails, but when bacterial cells cover the tracks they can pile more readily into these tracks resulting in phase dark trails. Track formation also revealed that occasionally the comets could move outwards whilst seeding no/few cells behind themselves (Fig. S2B). This behaviour may explain why microcolonies can sometimes be observed outside the main colony and also why these microcolonies can be arranged in straight lines as the *S. aureus* comet would be depositing only a few bacterial cells at distinct points (Fig. S2C).

### *S. aureus* colonies avoid each other and produce a surfactant halo

**Figure 6:**
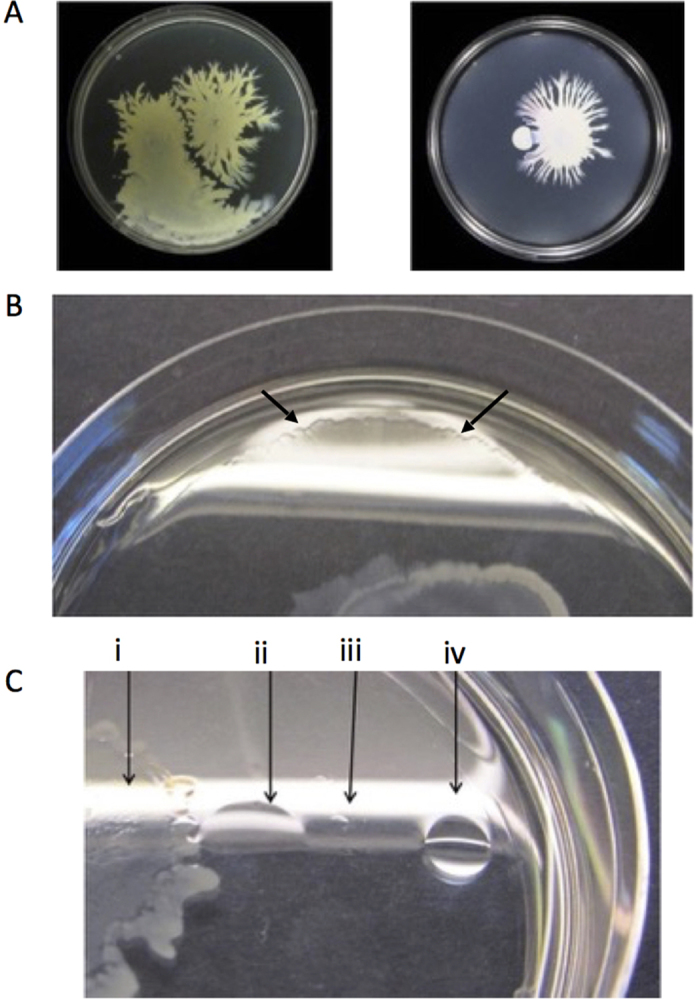
*S. aureus* engages in colony avoidance. (**A**) wildtype *S. aureus* colonies avoid each other (left panel) but an *agr* mutant (small colony) is not avoided by wildtype colonies (right panel) (**B**). wildtype colonies produce a halo of surfactant on the surface of the agar plate around the colony that can be observed using oblique illumination, whereas *agr* mutants do not (not shown). The edge of surfactant is highlighted with arrows (**C**). drop collapse test demonstrating that the halo is surfactant: (i) main colony, (ii) water droplet collapses due to surfactant action within the surfactant halo, (iii) the edge of the surfactant halo, (iv) a water droplet does not collapse outside the surfactant halo.

We then investigated whether *S. aureus* colonies could avoid each other as this is a behaviour associated with active swarming motility, where the bacterial cells also group together to move^16^,^24^,^25^. We found that they could avoid each other when spotted on a plate together (Fig. 6A), that wildtype colonies generally stopped short of colliding, and that dendrites at the periphery tended to turn away from both colonies. When wildtype and *agr* mutant colonies were paired, the wildtype colonies were not impeded by the *agr* mutant colonies and collided with them. A possible factor in this is the existence of a surfactant halo around the colonies as shown by oblique illumination (Fig. 6B) and the response to the surfactant drop collapse test (Fig. 6C). This surfactant halo was only found around the colonies formed by wildtype strains and not *agr* mutants (data not shown). Surfactant production has previously been observed in the original spreading assay and the surfactant is believed to consist of PSMs, whose expression is controlled by *agr*^17^,^26^,^27^. The surfactant zone varied in diameter from 1 mm to 1 cm away from the colony. It was always present at the same time as comet formation but typically disappeared after 24 h growth. We then investigated whether the movement of the comet head could be distinguished from the outward expansion of the surfactant halo or the movement of cells in the comet tails. We added a 5 μl drop of PBS as close to the comet as possible (after 8 h of incubation), and the surfactant present dispersed the droplet. As the droplet fluid dispersed outwards, it came into contact with the bacterial cells and pushed them away. We found that as the fluid expanded across the surface of the plate, it readily pushed away the bacterial cells in the comet tail but did not move the comet head, though cells were shed around the slime-covered section (Fig. S3).

## Discussion

### *S. aureus* comets appear to represent a form of active motility

We show for the first time that *S. aureus* on the surface of soft agar media can form structures colloquially called ‘comets’ whereby a group of cells advance across a surface seeding cells behind (Fig. 2B). More specifically, we found that (1) these comets preceded dendrite formation and are associated with colony expansion; (2) comets can move forwards, seeding cells behind themselves but without apparently losing mass (Video S3); (3) comet heads are held together by a slime matrix (Fig. 4); (4) comets can etch the agar under certain conditions (Fig. 5); (5) comet tips can move when no cells in the vicinity are moving, and at certain time points represented the only form of directional movement present (Fig. 3; Video S3); (6) no observable motility mechanisms such as flagella or pili were present, and (7) comets could not readily be moved by the addition of exogenous fluid (Fig. S3). As we will discuss, these results fit best with *S. aureus* being actively motile.

Actively motile bacteria are distinguished from passively motile bacteria by either, (1) observing previously identified characteristics that are associated with active motility and not passive motility (e.g. flagella), or (2) identifying situations where active propulsion can only be responsible for the observed movement of the bacteria (e.g. observing the bacteria swim). The identified characteristics of *S. aureus* comets, particularly slime, track formation, directional movement, lack of observable appendages and directed smooth movement are consistent with the characteristics of gliding motility^3^,^11^,^12^. However the gliding bacteria mainly studied at present (*Mycoplasma*, *Myxococcus xanthus* and *Cytophaga*) are actively motile as single cells (or move as single cells within larger groups as is the case with *M. xanthus* S-motility) and no *S. aureus* cells were observed to be actively motile as single cells. Certain filamentous bacteria have however been discovered, such as *Beggiatoa* and *Oscillatoria*, that were only able to glide as long chains of cells (which is also how they grow) and not as individual cells^12^. *S. aureus* may be similar, whereby cells move in a similar arrangement to which they grow, i.e as clusters of cells. The comet cores may also be formed of discrete groups of cells as this enables the round cocci cells to define a forward direction in which to travel based on the presence of the cells around them^28^, and aggregation is often associated with gliding^11^. Conversely, there may also be an as yet un-described way for individual *S. aureus* cells to be actively motile.

A second argument for active motility, can be shown via a physical analysis, such as the addition of fluid and observing the effect on the comet. For the comet to be explained as a passive form of movement, the comet head must have some special properties that means it moves more than the bacterial cells in the comet tail. It is either less firmly attached to the surface, or has a special mechanism allowing the surfactant around it to push it forwards preferentially. We excluded both of these possibilities by adding exogenous PBS to the surface near the comet, and examining how this moves the bacteria (Fig. S3). This is an acceptable test because surfactant works through facilitating the adsorption of water to a surface and reducing the surface tension around the bacteria^29^. By greatly increasing the supply of water, the bacteria should move more if surfactant expansion is the main source of movement. The comet heads moved remarkably little whereas the cells in the tail were scattered (Fig. S3). This is opposite to the effect expected if the comet heads were moving passively, and therefore it can be argued that active motility must be at least partially responsible for their movement. In addition, the firm attachment to the agar is consistent with the ability of the comet heads to etch the agar as this shows that the comet maintains tight contact with the agar (Fig. 5).

Conversely, our observations are not consistent with previously observed forms of passive motility such as spreading (sliding), darting or fluid carriage^5^,^10^,^30^. Darting looks physically different from what we have described, where projecting bacteria sporadically move very short distances, which is unlike the smooth movement observed in comets^3^. We did observe movement that appeared to be due to the bacteria being carried along by fluid (Video S1) (which we believe is surfactant), as many of the bacterial cells within the field of view were being carried along in the same direction and velocity, which does not occur when the bacteria are moving under their own locomotive force. This observation is similar to a combination of sliding motility and fluid carriage, in that surfactant enables movement like sliding motility but it is different as there is no even monolayer of bacterial cells present and the surfactant is carrying the bacteria within itself. This is characteristic of the previously described spreading motility^5^, which we believe should be more accurately termed sliding; our observations confirm its presence in this assay as well. Sliding motility and fluid carriage do not, however, explain comet formation as they cannot move large groups of cells (such as within a comet) without moving all the lighter groupings of cells around them at a faster rate (as mentioned above with the physical analysis). They also cannot account for the occurrence of slime around the comets alone and the etching of the agar by comets.

Is has been suggested to us that the motility we observed can be explained by purely physical effects. Such explanations focused on how the activity of surfactant can affect comet heads. These explanations include (1) two-phase liquid mixing effects (due to surfactant chemistry); (2) the Marangoni effect; (3) the bacteria in the comet head being able to roll; (4) the directed pushing of the comet head by cells growing behind it; (5) the comet head being able to move due to being surrounded by a hygroscopic matrix and surfing on the expanding surfactant; (6) changes in surface tension resulting from either physical or chemical conditioning. These explanations rely upon the comet head being weakly attached to the surface of the substrate (at least to the same extent as the bacteria in the comet tail) to explain the movement. We therefore tested these hypotheses by applying exogenous fluid to the comets, and showing that it did not preferentially move the comet heads (Fig. S3). Indeed they hardly moved at all, and so the above explanations based on the current literature can likely be excluded as the source of movement of the comet core. More generally, these phenomena when cited elsewhere, have been evoked to explain bacteria expanding on broad fronts^31^,^32^,^33^. The characteristics observed in association with comets (e.g. slime production, track formation) are not associated with passive forms of bacterial motility. It is possible that future research will demonstrate that comets can be formed and moved through passive forces, so we are basing our conclusions on the current state of the literature.

Overall we suggest that *S. aureus* can be actively motile under certain conditions, which is contrary to the previous belief that it is non-motile^4^. It is not surprising that *S. aureus* active motility has not been reported before. Active motility that is not dependent on observable appendages would be hard to initially detect. There is frequently little to observe when it is grown in liquid culture, and requires the right surface consistency and nutritional requirements to display motility on surfaces^12^. Additionally, active motility which is not mediated through flagella or type IV pili, has evolved separately in different bacterial species, which means identifying motility mechanisms through protein homology searches between different genera is generally not effective^13^,^14^. The form of motility that *S. aureus* engages in most closely resembles gliding motility, although we do not go so far as to define it as gliding.

### The role of surfactant in the motile colony

The surfactants that have previously been identified as important for spreading are the PSMs^6^,^17^. As *agr* directly regulates PSM production, this explains why *agr* is required for general motility of the colony (Fig. 1), and also likely contributes to comet movement as comets are not found without surfactant being present. Although *agr* and PSMs are important for virulence in their own right, it has previously been speculated that the original role of *agr* may have been to aid dispersal due to its close association with the biofilm dispersing PSMs^27^. Although bacteria can move using surfactants alone (sliding motility), their presence here does not preclude active motility, surfactant production is believed to be important for some forms of gliding motility and other forms of active motility such as swarming are also dependent on surfactant production in order to move^16^,^29^. The production of surfactant in connection with swarming motility is notably also commonly regulated via quorum sensing^16^. Additionally, the initial production of surfactant followed by the production of organized groups of bacteria that form the dendrites in *S. aureus*, resembles the timing of surfactant production and the lag in the formation of dendrites seen in swarming bacteria such as *Pseudomonas aeruginosa.* Furthermore, it is interesting to note that gliding motility in general has been described as flagella-negative swarming in the past^3^.

### Speculated motility mechanism and model of movement

If *S. aureus* is actively motile, then we can speculate on possible mechanisms based on our observations. In actively motile bacteria that lack flagella and type IV pili, a variety of mechanisms have been proposed as the means of propulsion: focal adhesion complexes, cell twisting, slime and membrane protrusion (in mycoplasmas)^9^,^13^,^34^. The coccoid shape of *S. aureus* cells and the absence of a slime trail and appendages, probably excludes cell twisting, pili, and slime extrusion as possible motility mechanisms. By a simple process of elimination, the motility mechanism powering *S. aureus* movement may be a focal adhesion complex (a membrane spanning mechanism which can reversibly attach to surfaces, as hypothesized in *Cytophaga* and *M. xanthus* A-gliding). However, it could easily be an as yet un-described mechanism^13^,^14^,^34^,^35^,^36^. We are suggesting this as a starting point for further research, rather than a definitive prediction of how *S. aureus* moves. A model of the comet formation behaviour is proposed. A *S. aureus* colony initially starts producing *agr-*dependent surfactant, and can generally expand outwards using sliding motility (spreading). After a few hours, some of the cells at the edge of the colony group together and move outwards as comets on the surfactant. These *S. aureus* comets use slime to hold the mobile group together, and move by an unknown but active mechanism. The comets can etch the agar as they move, and usually seed cells behind themselves. Bacterial cells that follow the comet tip stay within the tail (unless outside forces are present), as is seen with other bacterial species^15^. These may or may not be actively motile (they lack the defining features of active motility the comet head has) and this requires further investigation. The comets carry on moving until the plate becomes too dry, and then dissipate as comet formation is no longer effective, resulting in them disappearing after 24 h growth. The cells deposited by the comet grow into the dendrites we can observe with the naked eye on agar plates.

### Impact of these observations

Our results could impact a number of research areas. Showing that a pathogenic bacterial species is actively motile is important because motility mechanisms of all types have previously been shown to play an important role in virulence and colonization^1^,^37^,^38^. Therefore, a key next step could be to identify non-comet forming mutants and test their impact on virulence, as well as how the affected cells interact with each other, eventually generating a model of the motility mechanism. This is worth pursuing as motility mechanisms are considered reasonable targets for vaccines and inhibitory compounds^39^,^40^. As comet formation occurs across a range of *S. aureus* strains (Fig. 2B), it would seem likely that the underlying motility mechanism is well conserved in different *S. aureus* strains and so could present an attractive antimicrobial target. With all motility mechanisms, there has been a lag between the discovery of the motile behaviour and elucidation of the mechanism(s) involved so the process of its discovery in *S. aureus* remains a significant challenge^13^,^34^,^36^. Additionally, if *S. aureus* motility turns out to be important for virulence, this may stimulate discussion about how *S. aureus* initiates disease. In a large proportion of *S. aureus* bacteraemias, the entry point of infection is never found and it is generally assumed that it is too small to find^41^. It may be the case that *S. aureus* can use motility, and its suite of virulence factors, to enter hosts without obvious sites of entry and, furthermore, have increased dispersal across host surfaces in addition to simple attachment and passive dispersal alone, as is currently theoretically assumed. It may also be the case that motility is important for host or prosthetic material colonization as it is for other pathogenic organisms. Finally, if *S. aureus* is actively motile, it would also be the first example of a Gram-positive bacteria with a typical Gram-positive cell wall which can move without flagella or pili. Therefore, other Gram-positive organisms may also be motile in a similar and as yet undiscovered fashion.

## Materials and Methods

### Bacterial strains, media and growth conditions

All laboratory strains used in this study are described in Table 1. Single colonies of each strain were grown in 5 ml of Tryptone Broth at 200 rpm, 37 °C for 8 h. Required amounts of culture were centrifuged and re-suspended in Phosphate Buffered Saline (PBS) to an optical density of approximately 1.0 at 600 nm. This culture was then used to inoculate motility assay plates.

### Motility assay

The spreading assay previously developed by Kaito *et al.* was modified to incorporate elements that are used in the *P. aeruginosa* swarming assay^5^,^16^,^42^,^43^. This new assay, containing an increased agar concentration compared to the previously described spreading assay, was developed both to make the dendrites easier to observe under the microscope by making the media more stable, and also because agar concentrations above 0.3% are known to form solid surfaces, which excludes some forms of colony expansion and forces bacteria to move over the agar surface^16^. The modified spreading motility media used the following: 15 g of Tryptone Soy Broth (Oxoid), 1.7 g of Bacto agar (0.34% agar) (Oxoid) and 460 ml of distilled H_2_0. This was autoclaved, cooled to 55 °C for 30 mins and then used the same day. A Glucose solution was also prepared: 40 ml of water and 4.5 g of D-Glucose (Sigma) (50 mM), which was filter sterilized (0.2 μm filter, Millipore). This was added to the motility media and mixed just before the plates were poured. 25 ml of the combined media was added to 9 cm petri dishes (18 per batch), which were then dried for 30 mins in a type 1 safety cabinet, with lids inverted by their sides. Plates were then sterilized by placing them in a UV sterilization box for 10 min. 5 μL of bacterial culture was spotted onto the centre of a plate. Plates were then placed upright and unstacked in empty and dry plastic boxes and the boxes sealed (to maintain relatively even humidity and to control evaporation) in a 37 °C incubator overnight. The motility assay was further modified to make it suitable for certain observations by preparing the plates using 10 ml of media and drying them for 10 minutes, otherwise all other details were the same.

### Phase contrast microscopy

Motility plates were observed after incubation overnight using a Nikon Eclipse TE2000 inverted phase contrast microscope. All plates were examined at ×100 magnification and sample images taken using the integrated Nikon DXMI200 Camera. When taking ×400 magnification images, the modified 10 ml assay was used.

### Physical testing of the *S. aureus* heads

Motility plates were observed using a Nikon Eclipse 50i phase contrast microscope at 100× magnification after 8 h. A 5 μl drop of Phosphate Buffered Saline was added as close to a comet as possible and the fluid was observed as it spread out over the comet.

### Time-lapse microscopy

A DMIRB microscope (Leica) with a heated cabinet was used to generate time lapse videos. The heated cabinet could not accommodate 9 cm petri dishes so 6 cm petri dishes were used. The motility assay was revised to the following specifications for this technique: 5 ml of motility media (15 plates per group) was dried for 10 min. After UV sterilization and spotting of culture, the plates were placed in a cold room overnight (4 °C). The plates were then transferred to a 37 °C incubator for 4 h in the morning. Plates were then transferred to the microscope, which was maintained at 35 °C (the heating was inefficient), where they were observed using a ×100 objective and one frame was generated per second. The speed of the generated videos (7 frames a second) was increased 8 fold using Premiere elements (Adobe™) and therefore 32 seconds of video is equivalent to 30 min of real time (56 frames a second).

### Environmental Scanning Electron Microscopy (ESEM)

The ESEM stage would only take samples <2 cm^2^ in size so the assay was further modified: 5  ml of media per 9 Cm petri dish (with 5 minutes of drying) was poured over a 5 cm^2^ nitrocellulose 0.45 μM filter membrane backing (Millipore), all other parts of the assay remained the same. After 8 h, where the nitrocellulose backing was not present, the motility plates were observed for the necessary macroscopic structures using the phase contrast microscope to confirm their microscopic structure. The same macroscopic structures were observed on the nitrocellulose backing by holding them up to the light and then a 2 cm square was cut around these structures using a scalpel without disturbing them. These were then transferred to a Philips XL30 FEG ESEM where the ESEM analysis was performed.

### General Techniques

Photos of the motility plates were taken using a darkfield background and a Canon Powershot SX 220 HS^44^. Surfactant detection was performed by spotting 10 μl of distilled water onto motility plates at various points as per the previously described drop collapse test^45^.

### Bioinformatic searches

Homology searches for motility associated proteins were performed using BlastP (Basic Local Alignment Search Tool - Protein) on its standard settings^23^. The following was compared against the *S. aureus* genome: FliC from *Bacillus cereus* E33L (flagellin, flagella filament protein), PilA from *P. aeruginosa* (Pilin, type IV pili filament protein), SpaA from *Corynebacterium diphtheriae* (Gram-positive fimbriae)^46^, AgmU and AglZ from *Myxococcus xanthus* (Gliding mechanism associated proteins that are hypothesized to make up the focal adhesion complex)^47^, RomR, MglA and MglB from *M. xanthus* (conserved regulators of motility)^48^.

## Additional Information

**How to cite this article**: Pollitt, E. J. G. *et al.* *Staphylococcus aureus* forms spreading dendrites that have characteristics of active motility. *Sci. Rep.* **5**, 17698; doi: 10.1038/srep17698 (2015).

## Supplementary Material

Supplementary Video S1

Supplementary Video S2

Supplementary Video S3

Supplementary Information

## Figures and Tables

**Table 1:** Bacterial strains used in this study.

Strain	Characteristics	Origin/Reference
Newman	Laboratory strain, *agr* group I	49, 50
Newman Δ*agr*::*ermB*	Newman∆*agr::ermB*, erm^R^	51
RN6390B (WT)	Standard laboratory strain, *agr* group I, Derived from RN6390, Reisolate from a RN6390 batch that had become mostly Δ*agr*.	51, 52
SH1000	Laboratory strain, *agr* group I Repair of 8325-4 strain *rsbU* deletion	53
SA564	Invasive isolate, *agr* group II	54
Na129	*agr* group III	A. Cockayne (personal collection)
*S.aureus* 18	*agr* group IV	A. Cockayne (personal collection)
USA 300 (JE2)	Nebraska library strain, agr group I	55
