# Supplementary material for: Staphylococcus aureus forms spreading dendrites that have characteristics of active motility
Source: Sci Rep. 2015 Dec 18;5:17698. doi: 10.1038/srep17698 (PMC4683532; doi:10.1038/srep17698)
Supplement: Supplementary Information [file srep17698-s4.pdf]

Supplementary information

Staphylococcus aureus forms spreading dendrites that have characteristics of active motility

Eric J.G. Pollitt,[a,b] Shanika A. Crusz,[a] and Stephen P. Diggle[a]

School of Life Sciences, University Park, University of Nottingham, Nottingham, NG7 2RD, U.K[a]; Department of Molecular Biology and Biotechnology, Firth Court, University of Sheffield, Sheffield, S10 2TN, U.K[b]

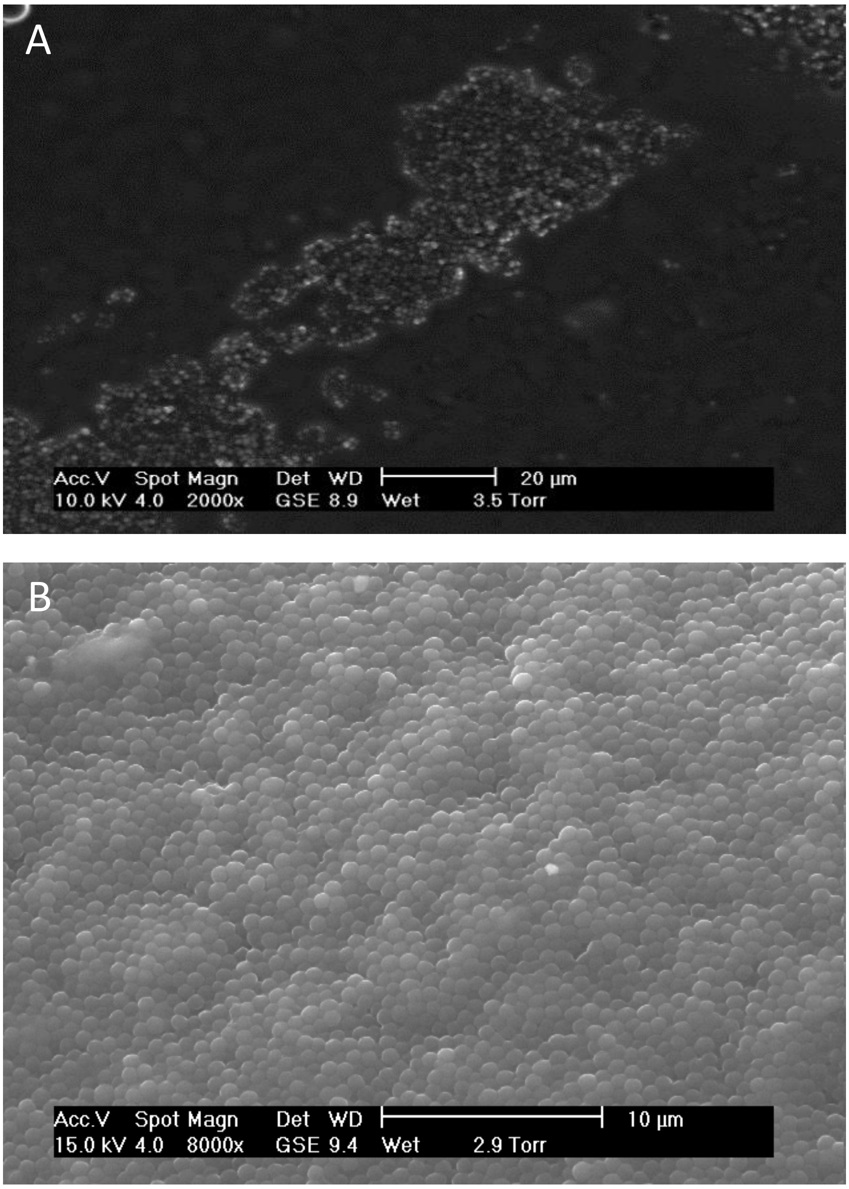

Figure S1. The comet cores are encased in a slime matrix. On searching the rest of the colony with ESEM, slime was not found outside comet cores, and the selective occurrence of slime has not been seen elsewhere with *S. aureus*. (A) *S. aureus* cells in the comet tail (from the same set of images as Fig. 4A). (B) *S. aureus* cells in the cen- tral mass of a spreading colony.

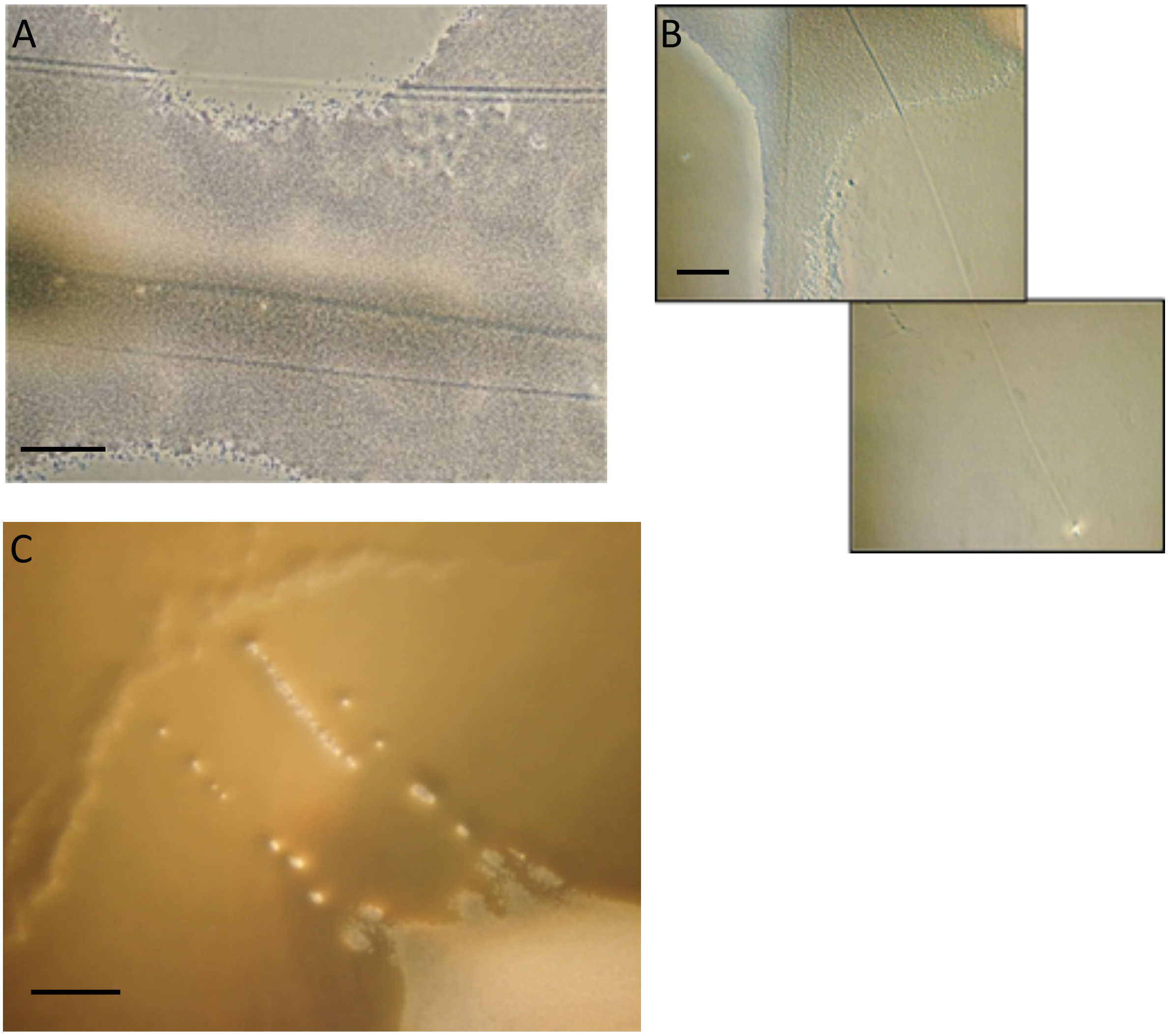

Figure S2. Comets can etch tracks in the agar. The etching of the media by the comets (Fig. 5) revealed other observations. (A) The tracks were phase bright when no bacteria were present and phase dark when they were present indicating that the tracks were most likely caused by the comets etching the agar. (B) The tracks reveal that sometimes comet cores can move whilst seeding no cells behind themselves and can provide an explanation why microcolonies can occur in lines running away from the main colony (bacteria seeded only intermittently). (C) Occasionally *S. aureus* micro-colonies occur, running in straight lines away from the central colony. A possible ex- planation for this is the occurrence of a comet which seeds few bacteria behind it, be- cause we have observed comet cores which do not seed any bacteria behind them (see Fig. S2B). All scale bars are 100µm.

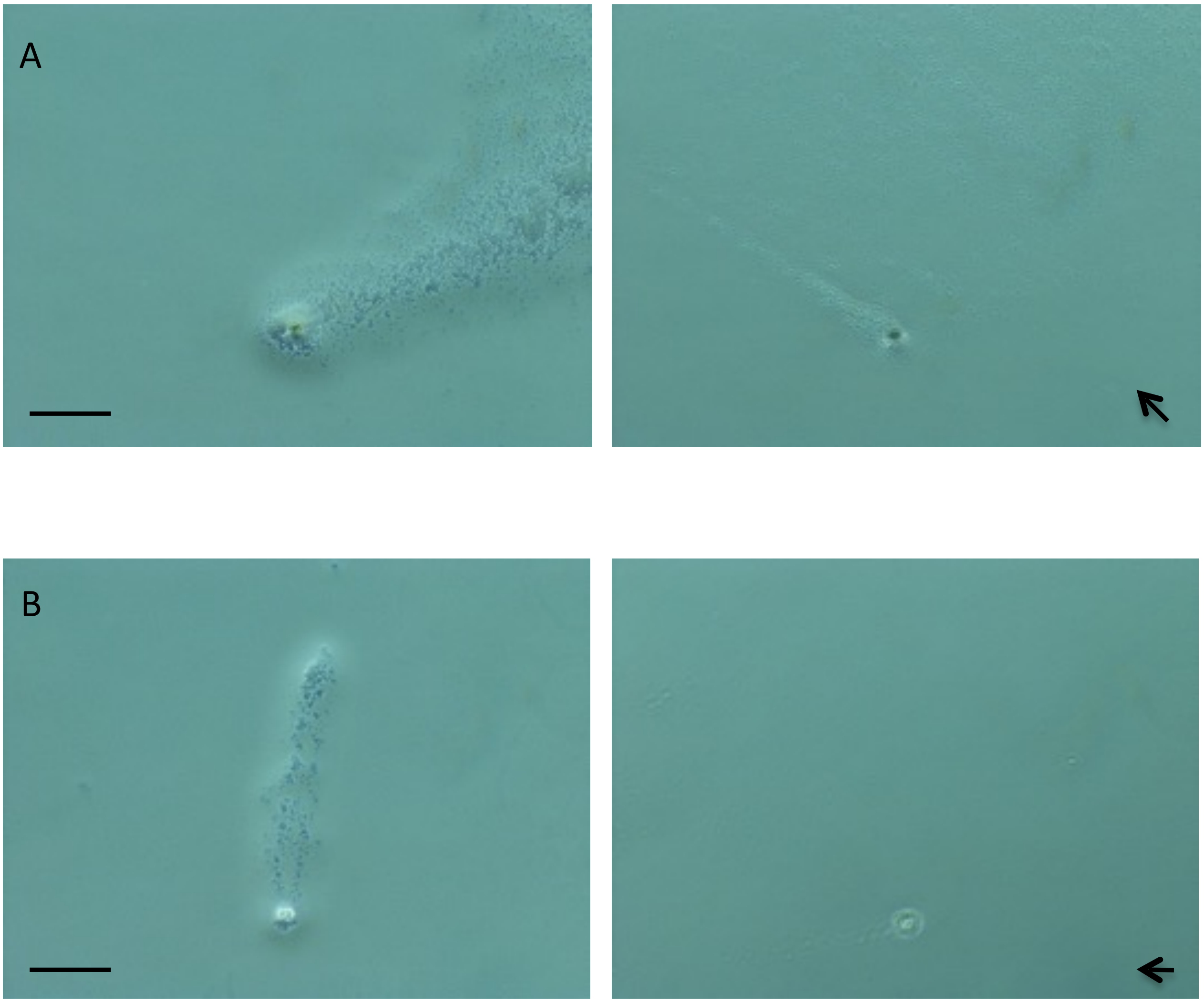

Figure S3. *S. aureus* comet heads are not readily moved by the addition of fluid. (A) In the left image a *S. aureus* comet is shown before the addition of 5µl of PBS. In the right image is the same comet after the addition of 5µl of PBS showing how the bacterial cells in the comet tail are readily scattered whilst the comet head stays relatively still. The arrow shows the direction from which the PBS was added. Scale bar = 100µm. (B) A second comet treated in the same way. Left image is before, and the right image is after PBS treatment.

Video S1. Timelapse video of passive motility on surfactant. This video shows how *S. aureus* cells can move on surfactant in a similar way to sliding motility. Bacterial cells are drifting in the same direction at roughly the same speed. We generated this video using a light microscope with a heated stage and a stabilized microscope table. 32 seconds of video is equivalent to 30 min of real time (56 frames a second).

Video S2. Timelapse video of *S. aureus* comet formation 5-8h. This video of comet formation was developed at a time point when the sample plate was wetter and surfactant generation was sufficient to move the bacteria alone. We generated this video using a light microscope with a heated stage. Here the comet moved quickly but the cells are eventually dispersed by a wave of surfactant entering at right angles. The surfactant wave more readily moved the bacteria in the comet tail than in the comet head and reveals that the comet head is adhered together more firmly than the bacteria around it. The comet core moves and seeds large amounts of bacteria behind it without losing cohesion or mass. 32 seconds of video is equivalent to 30 min of real time (56 frames a second).

Video S3. Timelapse video of *S. aureus* comet formation post 8h. The S. aureus comet is associated with the only movement in the local vicinity. We generated this video using a light microscope with a heated stage. The comet core moves and seeds large amounts of bacterial cells behind it without losing cohesion or mass. Cells in the tail can move for a period of time behind the colony but then stop moving and are always confined within the tail. Photos of the same comet over time are shown in snapshot form in Figure 3. 32 seconds of video is equivalent to 30 min of real time (56 frames a second).
